# Supplementary figures and images for: Ginsenoside Rb1, salvianolic acid B and their combination modulate gut microbiota and improve glucolipid metabolism in high-fat diet induced obese mice
Source: PeerJ. 2021 Feb 3;9:e10598. doi: 10.7717/peerj.10598 (PMC7866888; doi:10.7717/peerj.10598)

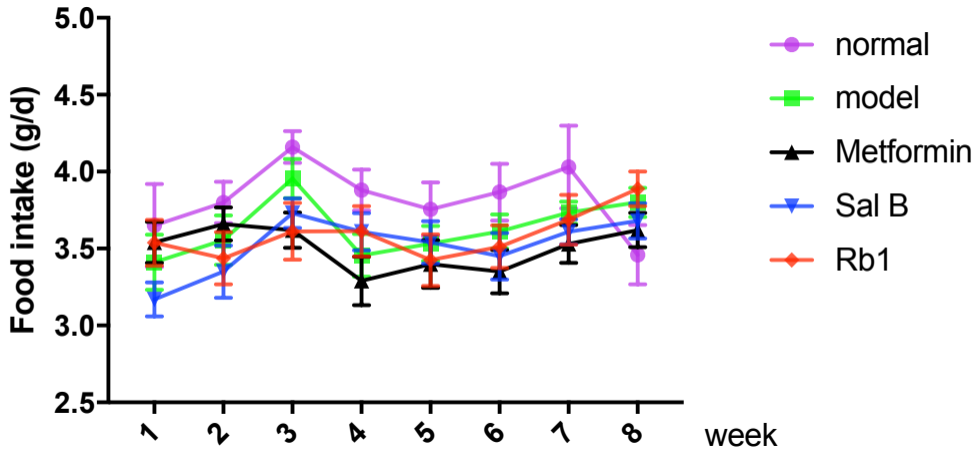

Supplement: Supplemental Information 2 [file peerj-09-10598-s002.pdf]

A

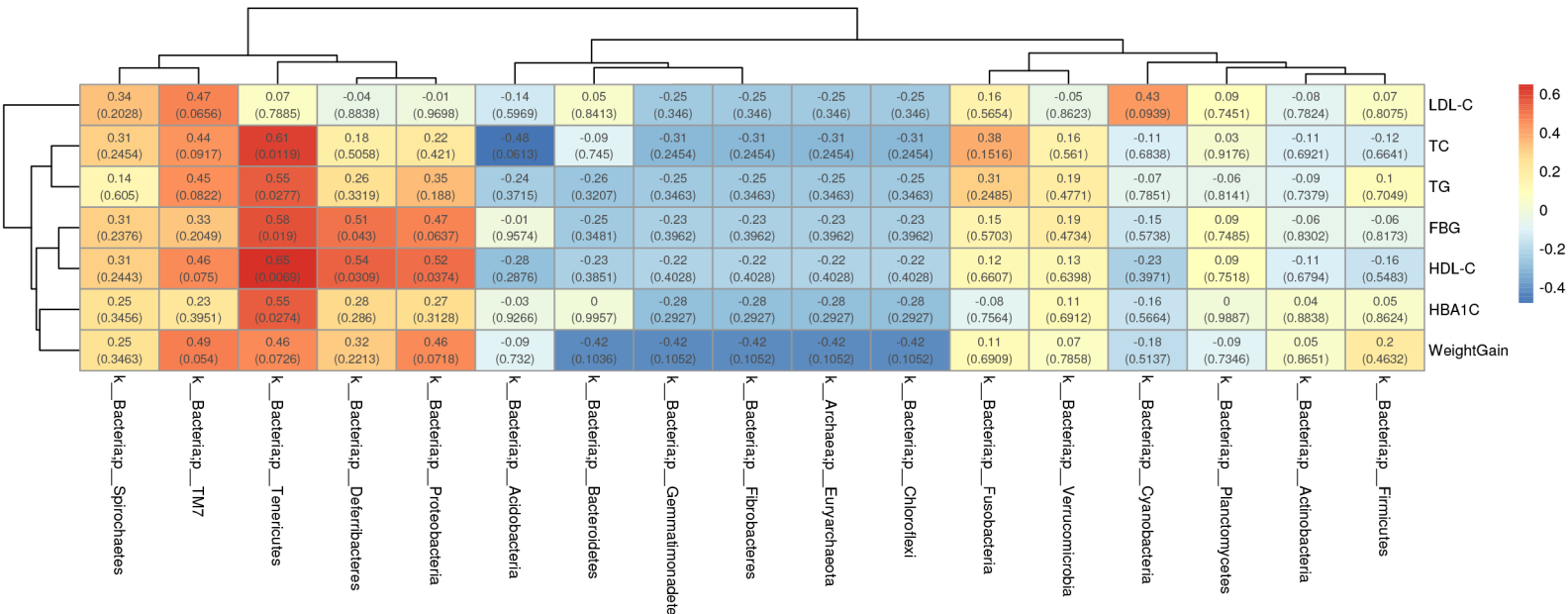

B

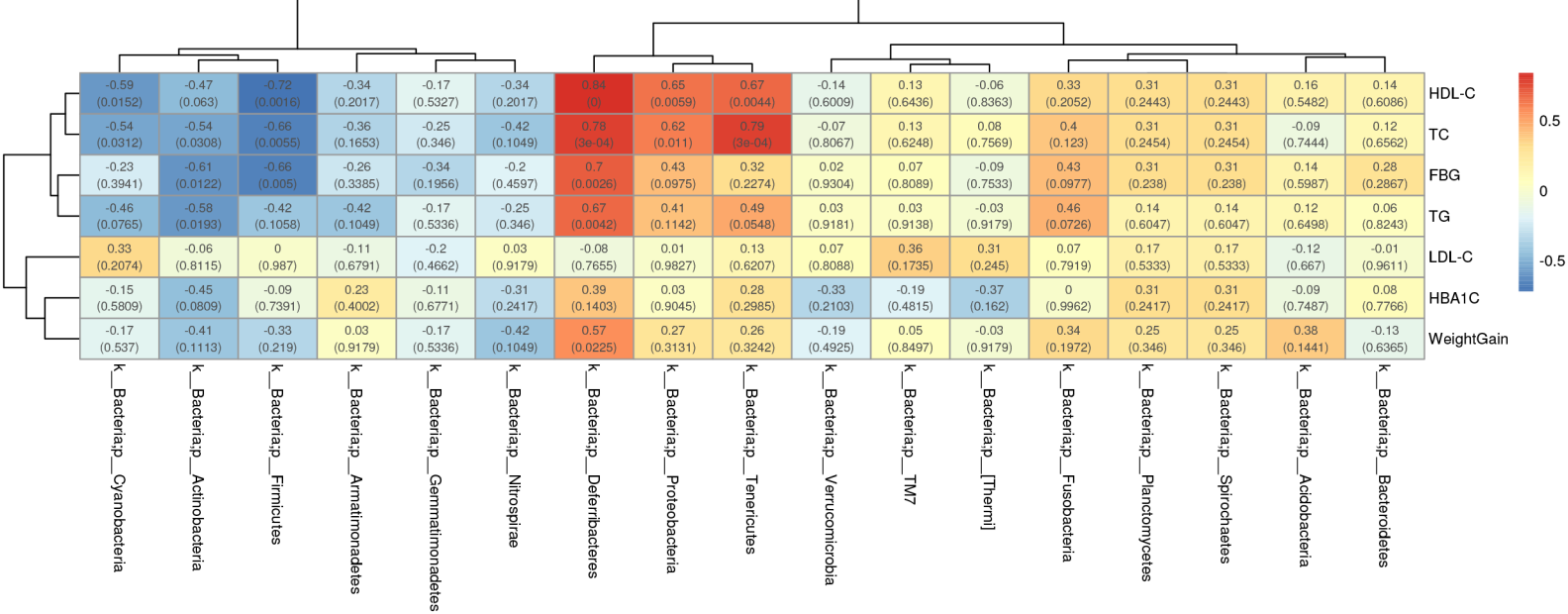

C

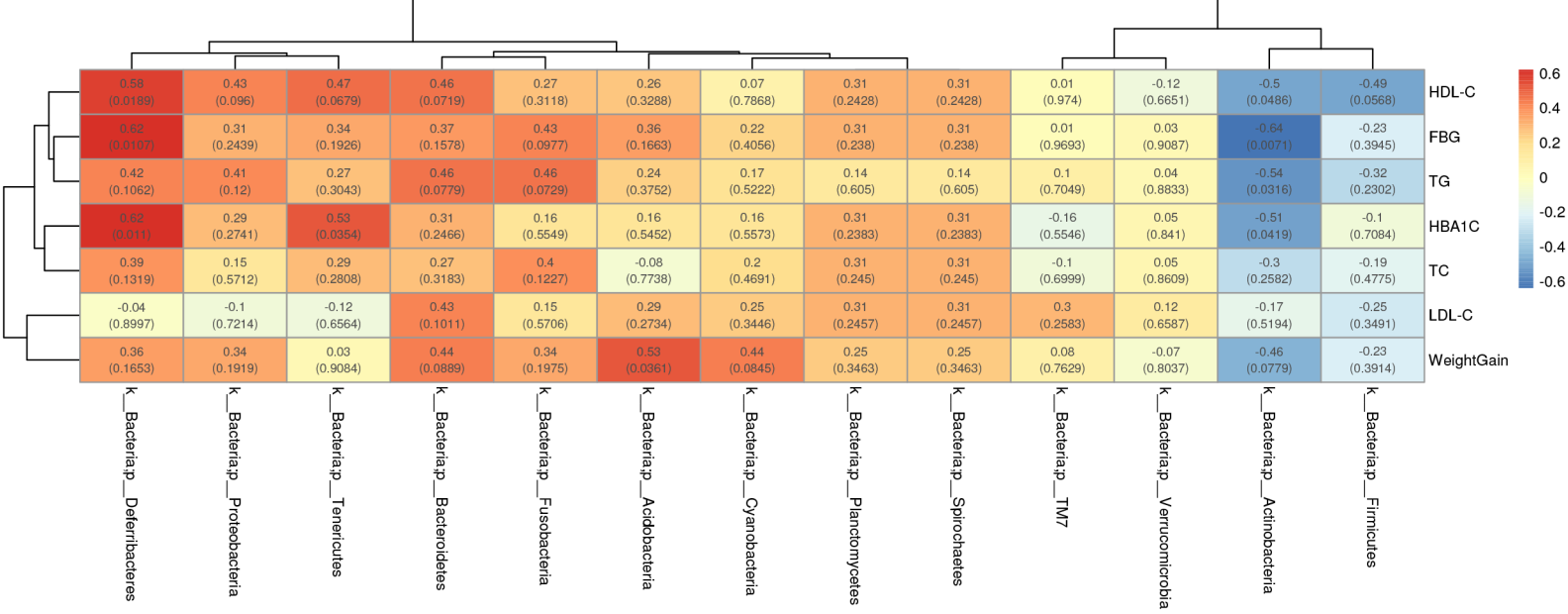

Supplement: Supplemental Information 3 — (A) The correlation heatmap of metabolic indicators and gut microbiota phylum between Con and Rb1 group. (B) The correlation heatmap of metabolic indicators and gut microbiota phylum between Con and SalB group. (C) The correlation heatmap of metabolic indicators and gut microbiota phylum between Con and Rb1SalB group. Note: each column in the graph represents a phylum, each row represents a metabolic indicator. The color in the graph indicates the Spearman correlation coefficient between the bacterial phyla and the indicators. Red represents positive correlation. Blue represents negative correlation. The darker the color, the greater the correlation. Number in the brackets is the P value. P < 0.05 is considered significant statistically. [file peerj-09-10598-s003.pdf]
