# Supplementary material for: Ginsenoside Rb1, salvianolic acid B and their combination modulate gut microbiota and improve glucolipid metabolism in high-fat diet induced obese mice
Source: PeerJ. 2021 Feb 3;9:e10598. doi: 10.7717/peerj.10598 (PMC7866888; doi:10.7717/peerj.10598)
Supplement: Supplemental Information 4 [file peerj-09-10598-s004.pdf]

Vs_group	SS	df	Ms	Fs	p-value
SalB-Rb1SalB	0.117286(1.34384)	1(1.34384)	0.117286(1.34384)	1.22188	0.141
SalB-Rb1	0.156304(1.38737)	1(1.38737)	0.156304(1.38737)	1.57727	0.015*
SalB-Con	0.151978(1.35311)	1(1.35311)	0.151978(1.35311)	1.57244	0.011*
Rb1SalB-Rb1	0.16763(1.29933)	1(1.29933)	0.16763(1.29933)	1.80617	<0.001*
Rb1SalB-Con	0.162372(1.26508)	1(1.26508)	0.162372(1.26508)	1.79689	0.001*
Rb1-Con	0.180782(1.30861)	1(1.30861)	0.180782(1.30861)	1.93407	0.001*
